# Phosphonium Salt-Functionalized β-Cyclodextrin Film for Ultrasensitive and Selective Electrochemical Impedance Spectroscopy Detection of Perchlorate in Drinking Water

**DOI:** 10.3390/polym17141937

**Published:** 2025-07-15

**Authors:** Zeineb Baatout, Achref Jebnouni, Nawfel Sakly, Safa Teka, Nuzaiha Mohamed, Sayda Osman, Raoudha Soury, Mabrouka El Oudi, Salman Hamdan Alsaqri, Nejmeddine Smida Jaballah, Mustapha Majdoub

**Affiliations:** 1 Laboratory of Interfaces and Advanced Materials, Faculty of Sciences, University of Monastir, Monastir 5000, Tunisia; zeinebbaatout@gmail.com (Z.B.); sakly_nawfel@yahoo.fr (N.S.); nejmeddine.jaballah@gmail.com (N.S.J.); mustaphamajdoub@gmail.com (M.M.); 2 Medical Surgical Nursing Department, College of Nursing, University of Hail, Hail 55476, Saudi Arabia; 3 The Higher Institute of Applied Sciences and Technology of Mahdi, University of Monastir, Rejich 5121, Tunisia; 4 Department of Chemistry, College of Science, University of Hail, Hail 55476, Saudi Arabia; 5 Department of Public Health, College of Public Health, University of Hail, Hail 55476, Saudi Arabia; 6 Chemistry Department, College of Science and Humanities, Shaqra University, Al Quwayiyah 11971, Saudi Arabia

**Keywords:** β-Cyclodextrin, phosphonium salt, perchlorate detection, water contaminant sensing, host–guest interactions, electrochemical impedance spectroscopy (EIS)

## Abstract

This work represents the first use of a phosphonium salt-functionalized β-Cyclodextrin polymer (β-CDP) as a highly selective sensing membrane for monitoring the safety of drinking water against perchlorate ions (ClO_4_^−^) using electrochemical impedance spectroscopy (EIS). Structural confirmation via ^1^H NMR, ^13^C NMR, ^31^P NMR, and FT-IR spectroscopies combined with AFM and contact angle measurements demonstrate how the enhanced solubility of modified cyclodextrin improves thin film quality. The innovation lies in the synergistic combination of two detection mechanisms: the “Host-Guest” inclusion in the cyclodextrin cavity and anionic exchange between the bromide ions of the phosphonium groups and perchlorate anions. Under optimized functionalization conditions, EIS reveals high sensitivity and selectivity, achieving a record-low detection limit (LOD) of ~10^−12^ M and a wide linear range of detection (10^−11^ M–10^−4^ M). Sensing mechanisms at the functionalized transducer interfaces are examined through numerical fitting of Cole-Cole impedance spectra via a single relaxation equivalent circuit. Real water sample analysis confirms the sensor’s practical applicability, with recoveries between 96.9% and 109.8% and RSDs of 2.4–4.8%. Finally, a comparative study with reported membrane sensors shows that β-CDP offers superior performance, wider range, higher sensitivity, lower LOD, and simpler synthesis.

## 1. Introduction

The prevention of drinking water pollution is an absolute priority that aims to save human life. Even though the conventional EPA analytical method (332.0) and currently available techniques including Accelerated Solvent Extraction (ASE) and Ion Chromatography-Mass Spectrometry (IC-MS) offer excellent sensitivity at trace level, these methods require cumbersome laboratory procedures and remain costly, labor-intensive, time-consuming, and especially unsuitable for on-site analysis. In response to these shortcomings, the current trend is the development of sensitive and selective methods allowing one to detect toxic compounds at ppm and ppb levels in aqueous media [1]. Perchlorate anion ClO_4_^−^ is one of the anions that present a high risk for the environment and a real menace to human health, as it interferes with iodine absorption by the thyroid gland and destabilizes hormonal functions [2]. These salts can release significant amounts of pressure, gas, or heat when subjected to elevated temperatures or exposed to an ignition source [3]. In 1998, the US Environmental Protection Agency (USEPA) considered the perchlorate ion as the most serious contaminant in drinking water [4]. It interferes with the absorption of the iodine by the thyroid gland, inducing a destabilization of hormonal functions. As a result, perchlorate ions can cause abnormalities in the development of children [5], disruption of thyroid gland function, and thyroid cancer in some cases [2,6]. Before 1997, significant difficulties in detecting perchlorate ions in groundwater at concentrations below 100 mg/L were observed. Since then, considerable efforts and huge budgets have been invested to improve the detection limit of perchlorate ions and to fix its maximum safe concentration in drinking water, which was determined to be 4 μg/L (40.2 nM) for newborns and 15 μg/L (151 nM) for adults by the California Department of Health (CDH) [5,7]. Most perchlorate salts have good solubility in water, and once dissolved, perchlorate ions will have important mobility with relatively high stability, which exceeds well over ten years. Many treatment techniques are used to identify and eliminate the perchlorate ions from water [8]. Researchers have also studied the behavior of these perchlorate ions during water treatment [9,10,11]. Perchlorate, a persistent and worrying contaminant, demands developing sensitive detection and removal strategies. Therefore, researchers are making important progress in developing sensitive detection and removal strategies for perchlorate by employing advanced techniques, such as chromatography [12,13], amine-crosslinked cotton [8], and Surface-Enhanced Raman Scattering (SERS) [14,15,16]. Beyond detection, crafting specific tools for perchlorate removal is crucial. Ion-selective electrodes, constructed using ion-selective membranes, provide a targeted approach, effectively identifying and measuring perchlorate among other environmental components [17]. This allows for precise removal strategies. Furthermore, Electrochemical Impedance Spectroscopy (EIS) emerges as a versatile tool for understanding perchlorate’s interactions with its surroundings [18,19]. EIS provides a unique perspective on the perchlorate problem. This innovative technique employs low-amplitude AC voltages swept across a range of frequencies. This capability proves essential for elucidating the interaction mechanisms between perchlorate and its surrounding environment, thereby informing the development of effective removal strategies. Another promising approach focuses on cationic Metal-Organic Frameworks (MOFs). With their intricate pore structures, these custom-designed materials can selectively capture perchlorate molecules, generating a distinctive signal that reveals their presence [20,21]. Beyond MOFs, chemists are exploring the potential of host-guest molecule structures, such as calixarenes [22,23], phthalocyanine [24,25], and β-cyclodextrin [26] complexes. β-CD, a cyclic oligosaccharide, is highly valued by scientists due to its biodegradability, biocompatibility, and non-toxicity, with a hydrophobic cavity and a hydrophilic exterior [27]. This unique structure, characterized by an anisotropic distribution of hydroxyl groups, which can be substituted with various other groups [28], along with its tunable host–guest interactions, serves as a privileged support [29,30]. These features enable the formation of inclusion complexes [31], control solubility [32], improve stability [33], and support its application in various potential fields. The use of cyclodextrin-based chemical sensors in the literature is relatively limited to detecting heavy metals and molecules whose complexation mode is based on the “Host-Guest” mechanism [34,35,36]. Owing to advancements in cyclodextrin modification chemistry, several cyclodextrin derivatives have been prepared and successfully applied as sensing active layers [37]. For instance, sulfonated β-cyclodextrin has been employed for the electrochemical detection of dopamine, achieving a sensitivity of 0.90 μA μM^−^^1^ cm^−^^2^ and a detection limit of 1 μM [38]. Gadhari et al. further reported the use of a sulfated γ-cyclodextrin–carbon nanofiber composite for the enantioselective determination of the multichiral drug moxifloxacin [39]. Despite this progress, the number of functionalized β-cyclodextrin derivatives used specifically for anion sensing remains limited [40]. Examples include sulfide anion (S^2−^) sensors with detection limits as low as 0.90 nM [41], amide-functionalized cyclodextrins that selectively recognize monophosphate anions [42], and ammonium-modified β-cyclodextrins used for the detection of phosphate and pyrophosphate ions [43]. These studies demonstrate the potential of tailored cyclodextrin derivatives for anion recognition. However, to the best of our knowledge, there are no previous reports on the selective detection of perchlorate anions (ClO_4_**^−^**) using cyclodextrin-based systems.

This gap highlights a significant challenge in the field: despite growing interest in cyclodextrin chemistry and advances in electrochemical transduction, the selective and ultrasensitive detection of perchlorate remains largely unexplored. By leveraging both the host–guest capabilities of β-cyclodextrin and rational chemical modification strategies, this work aims to address that gap. The present study introduces a straightforward and cost-effective synthesis of a novel β-cyclodextrin derivative functionalized with triphenylphosphonium groups, designed for use as a sensing layer in an electrochemical impedance spectroscopy (EIS)-based sensor. This sensor enables selective detection of perchlorate ions in drinking water at trace levels, offering a promising tool for environmental monitoring and water safety assurance.

This is accomplished by a proposed approach based on developing a simple and highly sensitive biosensor that combines detection via a “Host-Guest” mechanism with charged entities for the detection of perchlorate ions in drinking water, addressing a critical need for public health and environmental safety. This approach is above all cost-effective due to the affordability of β-cyclodextrin compared to its α- and γ-counterparts, the use of common solvents and reagents, and the low operational costs of label-free EIS detection [44]. Our study details the synthesis route for phosphonium salt of β-CD, investigates its structural, morphological, and physicochemical properties, and assesses its sensitivity and selectivity towards perchlorate anions at low concentrations, concluding with a comparative study in relation to previously reported techniques.

## 2. Materials and Methods

### 2.1. Chemicals and Materials

All chemicals were purchased from Sigma Aldrich (St. Louis, MO, USA), and used without purification: β-Cyclodextrin (β-CD) (≥97%), triphenylphosphine (TTP) (99%), bromine (Br_2_) (≥99.5%), N,N-dimethylformamide (DMF) (anhydrous, 99.8%), sodium methoxide (95%), dichloromethane (≥99.5%), hexane (≥95%), diethyl ether (≥99%), chloroform (≥99%), potassium phosphate dibasic (K_2_HPO_4_) (≥98%), potassium phosphate monobasic (KH_2_PO_4_) (≥99%), potassium perchlorate (KClO_4_) (≥99%), potassium nitrate (KNO_3_) (≥99%), potassium iodide (KI) (≥99%), potassium acetate (CH_3_COOK) (≥99%), isopropanol (≥99.5%), acetone (≥99.5%), tetrahydrofuran (THF) (anhydrous, ≥99.9%), ethanol (absolute, ≥99.8%), methanol (≥99.8%), dimethyl sulfoxide-d_6_ (DMSO-d_6_) (99.9 atom % D), chloroform-d (CDCl_3_) (99.8 atom % D), formamide (≥99%), diiodomethane (99%), nitrogen gas (ultra-high purity, 99.999%), sulfuric acid (H_2_SO_4_) (95–98%, for piranha solution), hydrogen peroxide (H_2_O_2_) (30%, for piranha solution), platinum electrode (99.9%), and deionized water (18.2 MΩ·cm).

### 2.2. Measurements

Nuclear Magnetic Resonance (NMR), Fourier Transform Infrared Spectroscopy (FTIR-ATR), Thermogravimetric Analysis (TGA), Differential Scanning Calorimetry (DSC), Atomic Force Microscopy (AFM), and contact angle measurements were used to characterize the β-CD derivatives. NMR Chemical shifts were referenced to the DMSO signal. FTIR-ATR Spectra were recorded between 500 cm^−1^ and 4000 cm^−1^. TGA Measurements were conducted under nitrogen at 10 °C/min. DSC: Thermograms were obtained under ambient atmosphere at 10 °C/min from −20 °C to 250 °C. AFM was used in tapping mode with a gold surface. Contact Angle: DI water, formamide, and diiodomethane were used as liquids and measurements were averaged from three separate drops. Phosphate-buffered saline (PBS) solution (10 mM) was prepared using K_2_HPO_4_ and KH_2_PO_4_.

### 2.3. Synthesis

#### 2.3.1. Per-Bromide β-Cyclodextrine (β-CDBr7)

**Scheme 1:**
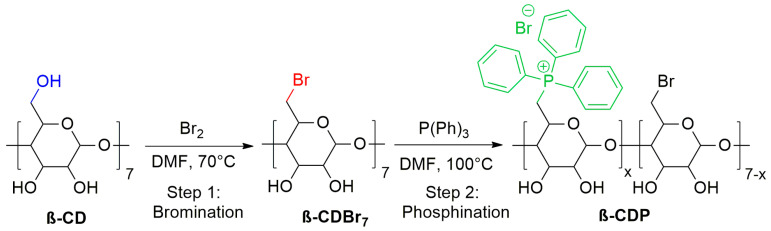
Synthesis route of the β-CDP.

The direct bromination of β-cyclodextrin was performed following the procedure described by Gadelle and Defaye [45]. The bromination reaction of β-CD was carried out in situ by nucleophilic substitution of the Vilsmeier bromide-activated primary alcohols. Triphenylphosphine and bromine Br_2_ were dissolved in N, N-dimethylformamide. After 3 h, β-CD was added to this solution and maintained at 70 °C for 15 h (Scheme 1). The reaction mixture was then treated with sodium methoxide to destroy the formed formats. The mixture was cooled, and the pH was kept below 9. Then, it was precipitated in an ice-water bath and filtered to remove sodium bromide. The residue was then washed with dichloromethane, which removes the formed triphenylphosphine oxide. A white powder was obtained with a yield of 90%. ^1^H NMR (300 MHz, DMSO-d_6_): *δ* = 3.28 (7H, t), 3.34–3.48 (14H, m), 3.57–3.68 (14H, m), 3.80 (7H, dd), 4.99 (7H, d), 5.91 (7H, d), 6.00 (7H, d), ^13^C NMR: *δ* = 18.0 (C-6), 70.9 (C-2), 71.8 (C-3), 72.1 (C-5), 85.9 (C-4), 102.0 (C-1).

#### 2.3.2. Phosphonuim-Modified β-Cyclodextrine (β-CDP)

β-CDBr_7_ (0.3 g, 0.19 mmol) was dissolved in DMF (5 mL) and heated at 100 °C under an argon atmosphere. Triphenylphosphine TTP (2 g, 7.62 mmol) was added by fractions to control the reaction rate, and the mixture was stirred for 48 h (Scheme 1). After cooling, the solvent was concentrated, precipitated into hexane, and washed several times with diethyl ether to give a beige powder. Yield: 53%. NMR ^13^C (CDCl_3_, 75 MHz, *δ* ppm): 35.0 (C-Br), 58.6 (C-2), 74.8 (C-3), 74.0 (C-5), 79.9 (C-4), 81.2 (C-1), 128.4- 134.7 (C-P) (Figure S2). NMR ^31^P (CDCl_3_, 121 MHz, *δ* ppm): 22.0 (d, *J* = 54 Hz) (Figure S3).

#### 2.3.3. Transducer Functionalization

In sensing experiments, a multi-layered structure of SiO_2_/Si/Ti/Au gold electrodes (surface area of 0.3 cm^2^) were employed as electrochemical transducers. Initially, the gold electrode surface was squarely cleaned and activated for 1 min with piranha solution (H_2_SO_4_/H_2_O_2_, 3:1 *v*/*v*) according to the conventional procedure previously reported in the literature [46]. Subsequently, the activated transducer was thoroughly rinsed sequentially with distilled water, isopropanol, and acetone. Finally, the surface was gently blown with nitrogen flow for drying. Surely, the stability of the active layer in contact with water remains the most relevant parameter for sensing applications. In attempts to target higher film quality and best adhesion stability in an aqueous medium, three surface functionalization methods were tested using a solution concentration of β-CDP (5 mg/mL) in chloroform. These included spin coating and dip coating methods with different speeds ranging, respectively, from 1000 rpm to 4000 rpm and from 5 mm·s^−1^ to 20 mm·s^−1^, as well as an incubation method for various periods of time. To overcome any probable structural change taking place in the sensing layer during thermal treatment, all the functionalized gold surfaces were subjected to a prolonged drying process at room temperature in normal laboratory conditions.

#### 2.3.4. EIS Spectroscopy

EIS spectroscopy measurements were conducted in a 25 mL three-electrode cell system in PBS buffer (10 mM, pH 7). While the employed working electrode was the functionalized gold electrode with the β-cyclodextrin phosphonium derivative, saturated calomel electrode (SCE) and platinum (Pt) were used as reference and auxiliary electrodes, respectively. AC impedance analysis was performed with an Autolab PGSTAT30 Potentiostat/Galvanostat controlled by the FRA software (Metrohm) for data acquisition using the Frequency Response Analyzer (FRA) software (Version 4.9.007, Eco Chemie B.V., Utrecht, The Netherlands). In experiments, DC bias potential and AC modulation signal amplitude were set to −300 mV and 10 mv, respectively, and the frequency range spanned 50 mHz to 100 KHz.

#### 2.3.5. Analysis of Real Water Sample

To evaluate the practical applicability of the proposed electrochemical sensor under real conditions, its performance was tested in complex water matrices. Tap water samples were collected from both the Faculty of Science of Monastir (University of Monastir, Tunisia) and from a household tap after allowing the water to run for 3 min. Additionally, 2 commercially bottled drinking water samples were randomly purchased from a local market. All collected water samples were spiked with different concentrations of the target analyte (ClO_4_^−^) without any prior purification. Each experiment was performed in three independent replicates (n = 3). The accuracy of the analytical method was assessed using the standard addition method, and the recovery percentage (%) was calculated using the following conventional formula:

(1) $$Recovery\left(\%\right)=\frac{Found\;concentration\;of\;ClO_{4}^{-}}{Added\;concentration\;of\;ClO_{4}^{-}}$$

#### 2.3.6. Computational Analysis of Data

Statistical analyses, including the calculation of mean values, relative standard deviation (RSD), limit of detection (LOD), and limit of quantification (LOQ), were performed using Microsoft Excel software. Additionally, data fitting was carried out using OriginLab software (version 7.0) with the Levenberg–Marquardt algorithm. The sensing mechanism was further analyzed through the simulation of an optimized Electrical Equivalent Circuit (EEC), obtained by using numerical fitting of impedance spectra using ZView^®^ software (version 2.09, Scribner Associates, Inc. (Southern Pines, NC, USA)).

## 3. Results

### 3.1. Synthesis and Structural Characterization

The phosphonium salt of β-cyclodextrin (β-CDP) was synthesized in two steps: bromination of β-cyclodextrin followed by phosphination of the resulting intermediate (β-CDBr_7_). The second step was carried out in DMF at 100 °C and yielded β-CDP as a fine powder (Scheme 1).

β-CDBr7 exhibited good solubility only in DMF, whereas the β-CDP was highly soluble in DMF, CHCl_3_, THF, ethanol, and methanol but had lower solubility in chloroform, acetone, and dichloromethane (Table S1). The structures of the obtained products were confirmed using NMR and FT-IR spectroscopies. Figure S1 illustrates the ^1^H NMR spectra of β-CD, β-CDBr_7_, β-CDP. The absence of the OH-6 peak at 4.5 ppm in the β-CDBr_7_ spectrum, present in the β-CD spectrum, confirms the complete substitution of all the OH-6 hydroxyl groups by bromine. We also clearly observe a signal around 7.8 ppm in the spectrum of β-CDP which can be attributed to the aromatic protons of the triphenylphosphine groups. The examination of the ^13^C NMR spectra (Figure S2) reveals a chemical shift of the peaks for the Phosphonium salt derivatives as expected (128.4–134.7 ppm). The ^31^P NMR (Figure S3) shows the presence of a doublet around 23 ppm corresponding to the C-P coupling ^1^J = 54 Hz (More details of the NMR study and the attribution of the different signals are given in the experimental section).

The degree of substitution of β-CD with the phosphonium group (DS) was determined using Equation (2) by comparing NMR integrals of phosphonium aromatic protons (3 phenyl rings × 5H each = 15H per group) and β-CD secondary OH protons in positions 2 and 3 (7 glucose units × 2 OH groups = 14H per molecule).

(2) $$\Rightarrow DS=\frac{14}{15}\frac{A1}{A2}$$

where

- *A*1 = integral of aromatic protons (7.8 ppm, 15H per phosphonium group).
- *A*2 = integral of OH-2/OH-3 secondaries protons (5.5–5.8 ppm, 14H) (Figure S1).

Therefore, the factor 14/15 corrects for the proton-count disparity. A DS was found to be 3.5 indicating ~50% substitution of OH-6 sites [47].

The FT-IR spectrum of β-CD (Figure S4) reveals the characteristic bands of the hydroxyl groups around 3400 cm^−1^, the aliphatic C–H stretching band at 2920 cm^−1,^ and the C–O–C stretching bands of the glycosidic bond at 1030 cm^−1^ [48]. The IR spectrum of the phosphonium derivative reveals a new weak absorption band at 3060 cm^−1^ corresponding to the elongations of the aromatic C–H bond and two bands between 1440 cm^−1^ and 1509 cm^−1^ corresponding to the elongation of the C=C bonds. The two bands with medium intensities appear at 782 cm^−1^ and 665 cm^−1^, which are attributable to aromatic C–H out-of-plane bandings. On the other hand, while the C–Br stretch at 583 cm^−1^ remains, its intensity diminishes in the β-CDP’s spectrum, suggesting a partial substitution of the β-CDBr7 ring.

### 3.2. Thermal Analyses

Thermal properties of the materials were studied using thermogravimetric analysis (TGA) and Differential Scanning Calorimetry (DSC). TGA thermograms (Figure S5) revealed a decrease in the degradation temperature (Td) from ~290 °C for the pristine molecule (β-CD) to significantly lower values upon bromination (220 °C) and phosphination (195 °C). This decline is likely due to substituting strong original C-O bonds with weaker new C-Br and C-P ones [49] and the induced disruption of the intramolecular hydrogen bonding networks. The DSC thermogram of β-CDP (Figure S6) showed no crystalline or melting peaks, indicating that the material is totally amorphous.

### 3.3. Thin Film Surface Properties

The morphology of thin layers created by incubating β-CD and β-CDP at room temperature was examined through Atomic Force Microscopy (AFM) and contact angle measurements conducted on gold substrates. Given the limited film-forming properties of β-CD, significant AFM images could not be obtained for the pristine molecule [50]. It is important to note that the inability to obtain significant AFM images for pristine β-CD is itself evidence of the dramatic improvement in film-forming properties achieved through our phosphonium salt modification. The unmodified β-CD did not form a continuous layer that can give significant AFM images, preventing direct quantitative comparison of surface roughness between β-CD and β-CDP. In contrast, the AFM images of the phosphonium salt derivative (Figure S7) reveal a marked enhancement in surface morphology, characterized by a relatively smooth texture and a Root Mean Square (RMS) roughness of 22 nm. The better film-forming ability and the smoother surface observed in the β-CDP film is attributed to the enhanced solubility of the derivative, which enables a more effective dispersion of β-CDP molecules on the gold surface compared to β-CD. This, along with π-stacking interactions between aromatic units of the phosphonium groups, promotes more uniform packing, leading to a consistent and even layer formation with minimal surface irregularities. To assess the surface polarity and wettability changes induced by β-cyclodextrin (β-CD) modification, we conducted a contact angle study using the Good Van Oss model [51]. Bare gold, with a moderate water contact angle (θ°) of 70°, is a reference point for hydrophobicity [52] (Table S2). Upon β-CD deposition, the water contact angle significantly drops to 46.4°, substantially increasing the basic energy component to 35.0 mJ/m^2^. This dramatic shift marks a transition to a hydrophilic character. β-CD’s abundant hydroxyl groups readily make important hydrogen bonds with water molecules through strong hydrogen bonding. This enhanced polar interaction overcomes the weak dispersive forces between water and gold, leading the water droplet to spread and wet the surface more readily. However, introducing triphenylphosphonium salt groups, which are less hydrophilic than hydroxyl moieties, reverses this effect, resulting in a β-CD derivative with water-repellent properties [53]. Consequently, the water contact angle for β-CDP climbs back to 73°. Indeed, Phosphonium salts in β-CDP slightly increase the acid energy component (γ+) while significantly decreasing the basic energy component (γ−). This interplay between hydrophilic and hydrophobic moieties demonstrates the tunable wettability of β-CD, opening doors for diverse applications tailored to specific surface properties and for enhanced stability and lifetime [47].

### 3.4. EIS Measurements: Preliminary Study

**Figure 1:**
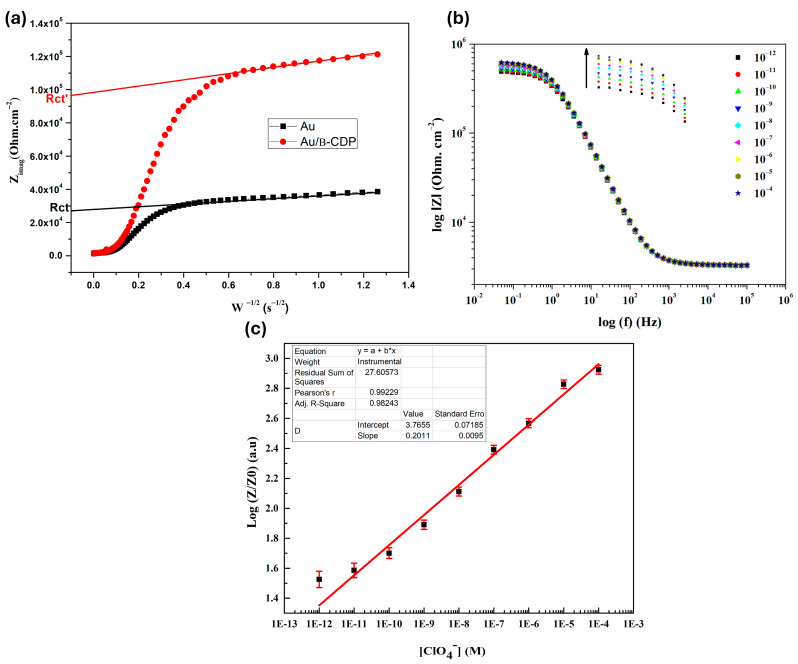
(**a**) Determination of surface coverage rates of the Au electrode. (**b**) Evolution of the impedance spectra (Bode diagram) as a function of the ClO_4_^−^ ion concentration. (**c**) Calibration curve of the β-CDP layer as a function of the cologarithm of the perchlorate ion concentration (Error bars in red color represent standard deviation from 10 independent measurements).

Initially, and in order to underscore the electrical effect of the deposited active layer, the direct overvoltage that can limit diffusion of electroactive species to the metal electrode/electrolyte interface [54] was optimized. Overall, the Cole-Cole spectra recorded for different DC bias potentials displayed in Figure S8 reveals concomitantly a net gradual disappearance of the Warburg straight line at low frequencies and a net decrease of the half-circle diameter at high frequencies. Accordingly, better control of the charge transfer at the interface Au electrode/electrolyte was gained for an overvoltage of −300 mV/ECS. The latter was chosen as the optimal DC bias potential for further experiments. Certainly, the functionalization method persists critically to ensure good reproducibility of measurements as well as a long lifetime of the sensor. Consequently, impedance measurements were performed on the bare gold electrode and three functionalized electrodes by mimicking operating conditions in the EIS experimentations (−300 mV DC bas potential, 10 mM PBS buffer, and neutral pH). The functionalized electrodes included spin-coated, dip-coated, and incubation-coated electrodes. The validity of the optimized functionalization method was evaluated by following the impact of the aging of the coated electrodes for 24 h on the evolution of the impedance spectra while comparing them to that of the bare electrode. Nevertheless, neither spin nor dip coating methods with different speed values, ranging, respectively, from 1000 rpm to 4000 rpm and from 5 mm·s^−1^ to 20 mm·s^−1^, produced good adhesion and the required stability for our intended application since their impedance spectra barely differ from that of the uncoated transducer after 24 h of immersion in PBS buffer solution. In contrast, whatever the incubation time is, good reproducibility of measurements was attained within the limit of the experimental error even after 24 h of aging. Consequently, the incubation was selected as the optimal functionalization method for further sensing experiments. Unambiguously, the determination of the coverage rate (θ) of the electrochemical transducer is crucial to better understanding the compact or porous nature of the deposited layer allowing one to better understand the sensing mechanisms occurring at the different interfaces (metal/active layer/solution). In this context, the extracted real part of the impedance of the bare and functionalized Au electrode from the Cole-Cole Spectra (see Supplementary Data Figure S9) was plotted as a function of the inverse of the square root of the pulsation of the AC modulation signal. By virtue of the extrapolation in the linear zone to high frequencies (Figure 1a), we determined the equivalent resistance of the typical Randle’s circuit: electrolyte resistance (Rs) and charge transfer resistance (R_ct_). Owing to the low value of the former, the equivalent resistance of Randle’s circuit could be governed by the R_ct_ value, which endures highly sensitive to the porosity or compactness of the active layer. A coverage rate of 86% was obtained using Equation (3) [55,56], which allows us to conclude that the incubation functionalization method yielded an unexpectedly dense and compact layer.

(3) $$\theta =1-\frac{Rct\left(Bare\;Au\;electrode\right)}{R’ct\left(Functionalized\;Au\;electrode\right)}$$

θ is the coverage rate, and *R_ct_* and *R*’*_ct_* are the charge transfer of the Au electrode before and after functionalization, respectively.

### 3.5. Impedimetric Detection of Perchlorate (ClO4^−^) Anions

Owing to the high stability of the active layer as previously mentioned, we examined the global impedimetric response of the Au/β-CDP structure toward different concentrations of ClO_4_^−^ ranging from 10^−12^ M to 10^−4^ M in 10 mM PBS at neutral pH. Clearly, the recorded Bode diagrams, sketched in Figure 1b, exhibit a sigmoidal shape (inverted S-shape), regardless of the concentration of the target analyte. More deeply, in the whole range of the investigated frequency, two distinct zones were observed. A static zone, above 1 Hz, insensitive to the successive injection of perchlorate concentration, and a plateau dynamic zone at low frequencies (below 1 Hz) marked by a sharp increase in |Z| as the target anion concentration increased. Consequently, in the latter zone where the impedance variation is substantial, we selected 50 mHz as an operating frequency point to evaluate the sensing performances of the Au/β-CDP structure toward ClO_4_^−^ anions at neutral pH. For this purpose, we plotted the response curve (|Z|/|Z_0_| vs. −log ClO_4_^−^), displayed in (Figure 1c), where |Z| and |Z_0_| are the modulus of the functionalized Au electrode impedance in the presence and absence of perchlorate anions, respectively. In addition, we determined the sensing performances of the developed electrochemical sensor based on the β-CDP active layer, which are gathered in Table 1. These include sensitivity, linearity, linear range, limit of detection (LOD), and limit of quantification (LOQ). The latter were evaluated following the universal method mandated by the IUPAC according to the following equations:

(4) $$LOD=\frac{3.3\times \sigma }{S}$$

(5) $$LOQ=\frac{10\times \sigma }{S}$$

where σ is the relative standard deviation (RSD) of a blank sample in 10 replicates, and S is the sensitivity calculated as the slope of the impedimetric response in the linear range [57]. Additionally, in the aim to assess the reproducibly of the developed sensor, we evaluated the coefficient of variation (CV) of the electrochemical response according to the universal statistical method based on 10 independent measurements.

Overall, the developed sensor based on the β-CDP sensitive layer exhibits good sensitivity, reproducibility, and linearity, wide linear range, and high sensitivity to perchlorate traces since the limit of detection and quantification persist in pM. Importantly, the maximum safe concentrations of the perchlorate ions (ClO_4_^−^) established by the US EPA (40.2 nM for newborns and 151 nM) for adults fall well within the detection range of the method reported in this work.

### 3.6. Modelling and Sensing Mechanisms

**Figure 2:**
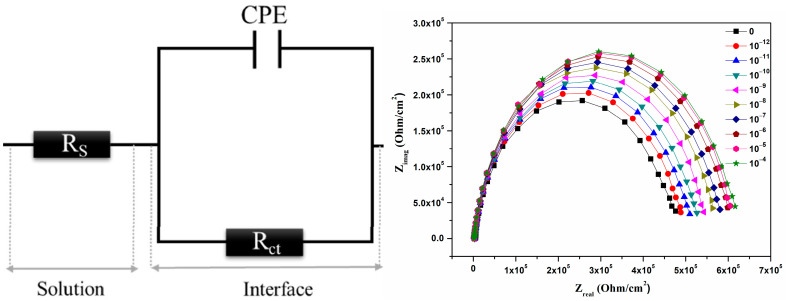
(**Left**) Equivalent circuit of Au/β-CDP/electrolyte interfaces. (**Right**) Variation of the impedance diagram of the Au/β-CDP structure as a function of the Z_real_ at different concentrations of the perchlorate ions.

To elucidate the electrochemical sensing mechanisms and probe the electrical contribution of the different interfaces, a modeling procedure was completed. Accordingly, the Randles circuit sketched in Figure 2 (left) was chosen as the optimized equivalent electrical circuit model. In fact, regardless of the tested concentration of ClO_4_^−^, the lack of asymmetry and distinguishable multi-capacitive semi-circles in the Cole-Cole impedance spectra profiles (Figure 2 (right)) simultaneously supported by the perfectly symmetrical bell shape of the phase curve in semilogarithmic scale depicted in Figure S10a suggests a single relaxation electrical model [58]. In the proposed EEC model, R_s_ is the resistance of the background electrolyte in series with a parallel dipole combining an R_ct_ resistance with a constant phase element (CPE). While the former reflects the electronic and ionic charge transfer at the metal/electrolyte, the latter is an extremely flexible electrical component to take into account the deviation from the Z_real_-axis of the center position of the capacitive half-circle. The CPE impedance expression is given by:

(6) $$Z_{CPE}=\frac{1}{Q(j\omega {)}^{n}}$$

where Q and n (0 < n < 1) are the CPE parameters. Noticeably, n = 0, n = 0.5, and n = 1, Z_CPE_ assumes the typical analytic expression of resistance, Warburg diffusion impedance, and ideal capacitance, respectively. Certainly, the lower the fidelity coefficient (χ^2^) is, better the correlation between experimental data points and the trend curves relating to the proposed EEC. For better clarity, Figure S10a,b displays an example of a simulation carried out using the optimized EEC for a particular perchlorate concentration of 10^−12^ M.

A good agreement between experimental results and the simulated Cole-Cole spectra using the complex nonlinear least square (CNLS) procedure was obtained since the parameter (χ^2^) persists lower than 10^−3^. Table S3 summarizes the different values of the electrical components of the optimized EEC model screws to screws of the cologarithm of the perchlorate concentration. The obtained fitting results clearly reveal a net increase of the global transfer charge resistance against a net decrease of the CPE element as the concentration of the perchlorate anion rises in the surrounding medium. Meanwhile, an index (n) value close to unity points unambiguously to a capacitive plane structure, which is supported by AFM investigation. Deeply, and referring to the ionic size of the ClO_4_^−^ anion which greatly exceeds that of bromide, the sharp decrease of the Q value is expected, allowing an inevitable increase of the effective thickness of the sensitive membrane. Moreover, the higher the charge density of the analyte and subsequently the ion polarizability, the more accentuated the increase of the dielectric permittivity is. However, considering the observed decrease of the Q value as a function of perchlorate concentration, a ‘Balance effect’ at the membrane/solution interface during the anion exchange between steric and polarization effects leads to an outweighing of the thickness effect against the effect of the variation of the dielectric constant. Such a trend promotes a higher surface rate coverage of the functionalized electrode, which is matched with the observed increase of the R_ct_ value. To highlight the contribution of each of the electrical components of the optimized EEC model in the proposed sensing mechanism, we artificially plotted in the same graph (Figure S11) the calibrations’ curves corresponding to the variation of R_ct_ and Q parameters toward the cologarithm of perchlorate anion concentration and evaluated the sensitivity and linearity (Table S4). The obtained results demonstrate clearly the predominance of the R_ct_ variation, indicating that the sensing mechanism is governed by the charge transfer process at the interface of the functionalized gold electrode/electrolyte.

### 3.7. Selectivity

**Figure 3:**
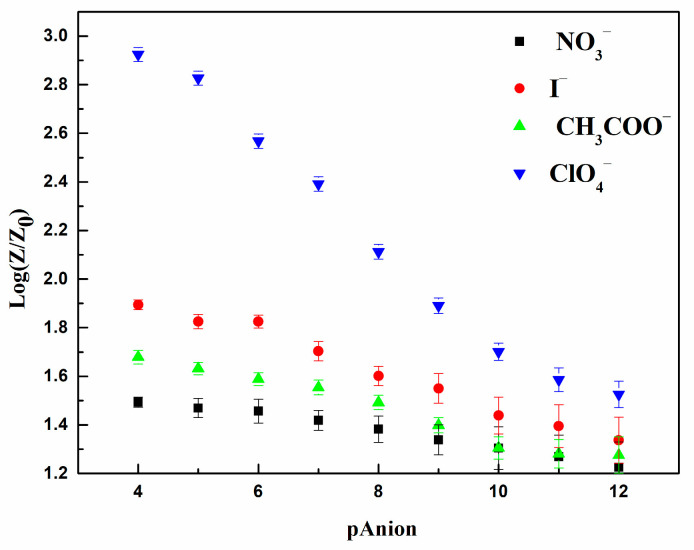
Response of the developed electrochemical sensor toward various anions in PBS (10 mM) at neutral pH (Error bars represent standard deviation from 10 independent measurements).

The selectivity of the sensitive membrane is crucial when used in real circumstances of employment given the variability of the chemical composition of drinking water. For this purpose, we determined the response of the sensitive layer toward nitrate (NO_3_^−^), iodide (I^−^), and acetate (CH_3_COO^−^) anions in the PBS electrolyte at a neutral pH mimicking that of drinking water. Figure 3 shows the variation of the impedance as a function of the cologarithm of the concentrations of the tested interfering anions.

In comparison with the sensitivity of the active layer toward perchlorate anion, a relatively weak impedimetric response for the triplet tested anions was detected, indicating a minor interfering effect. The obtained result is in good agreement with the selectivity classification according to the Hofmeister theory (ClO_4_^–^ → SCN^–^ → I^−^ → NO_3_^–^ → Br^–^ → Cl^–^ → HCO_3_^–^ → CH_3_COO^–^ → SO_4_^2–^ → HPO_4_^2–^) [59,60].

### 3.8. Analysis of Real Water Samples

The practical application of the developed electrochemical perchlorate sensor was investigated by detecting the target analyte in various water samples, including two locally commercialized bottled drinking waters and tap water samples. The accuracy of the proposed analytical method was evaluated by calculating the recovery at three different concentrations of the ClO_4_^−^ anion using the standard addition method, as presented in Table S5. In alignment with the guideline values set by the EPA and WHO, a concentration of 50 nM was deliberately included among the tested perchlorate levels. Based on the obtained results, satisfactory recovery values ranging from 96.9% to 109.8%, with relative standard deviations (RSDs) between 2.4% and 4.8%, were achieved across all tested samples. Accordingly, the proposed electrochemical sensor demonstrates the potential for accurate and reliable detection of ClO_4_^−^ ions in real water quality monitoring applications.

### 3.9. Comparative Study

A literature review was conducted on perchlorate ion detection methods, including impedance, potentiometric, capacitance, and optical studies, and we performed a comparative analysis between these methods and the technique used in our study; the characteristic parameters are summarized in Table 2. Compared to other developed membrane sensors based on organometallic complexes and cage molecules like calix[4]arene derivatives, the modified cyclodextrin (β-CDP) provides better linearity, a wider detection range, a good sensitivity, and a lower detection limit with a much easier synthesis method. These unique performances of the sensor result from the versatile combination of two synergistic detection mechanisms. First, an anionic exchange process occurs between the bromide ions of the phosphonium group and perchlorate ions, facilitating efficient ionic recognition. This is favored by perchlorate’s low hydration energy and high polarizability [61]. Second, the partial inclusion of perchlorate ion into the β-CD cavity, which is stabilized by chaotropic effects, where hydrophilic anions like perchlorate disrupt the water structure, makes encapsulation enthalpically favorable [62]. This dual interaction strategy merging anion exchange and host–guest chemistry demonstrates the potential of β-CDP as a highly effective and versatile platform for perchlorate detection, in line with emerging literature on advanced cyclodextrin-based sensing systems [42].

## 4. Conclusions

This study introduces a novel sensing platform based on a phosphonium salt-functionalized β-Cyclodextrin polymer (β-CDP), marking the first application of such a material for perchlorate detection in water. The unique dual-mode sensing mechanism combining host–guest inclusion with anion exchange sets this approach apart from conventional cyclodextrin-based sensors and enables superior analytical performance. The β-CDP membrane exhibits excellent selectivity, a broad linear detection range (10^−11^–10^−4^ M), and an ultra-low detection limit (~10^−12^ M). Its enhanced solubility and film-forming ability, confirmed through structural and surface characterizations, further contribute to its practical applicability. Real water sample analysis confirms its field relevance, with high recovery rates and low RSDs. Compared to existing membrane sensors, β-CDP offers a simpler synthesis route, improved sensitivity, and a more effective design strategy. Future work will focus on investigating the interaction mechanisms in polar solvents using NMR, determining stability constants, and evaluating other cyclodextrin derivatives for sensing and removal of perchlorate and related contaminants.

## Figures and Tables

**Table 1:** The sensing performances of β-CDP layer.

Sensitivity (a.u)	CV (%)	Linearity (%)	Linear Range (M)	LOD (10^−12^ M)	LOQ (10^−12^ M)
0.20213 ± 0.0095	3.65	98.243	10^−11^–10^−4^	2.23	6.75

**Table 2:** Comparison of the performance of the developed sensor with previously reported sensors in the literature.

Method	Electrode	Membrane/Molecular Probe	Sensitivity	Linearity (R^2^)	Linear Range (M)	Detection Limit (M)	Ref.
Impedance	Gold	β-CDP	0.215	0.997	10^−11^–10^−4^	2.23 10^−12^	Our work
	Gold	Co(II) MAPc	0.061	0.982	9.1 × 10^−10^−10^−3^	9.1 × 10^−10^	[19]
	PVC	Phosphonium calix[4]arenes	58 mV	-	5 × 10^−7^–10^−2^	10^−7^	[63]
Potentiometric	PVC	Polysiloxane ether couronne	-	0.993	6.3 × 10^−7^–10^−3^	4.6 × 10^−10^	[64]
	PVC	Hexaaza cyclohexa decane Cu (II)	59.4 mV	0.994	10^−6^–10^−1^	4 × 10^−7^	[65]
	PVC	CGE-Macrocyclic Zn (II)	58.7 mV	0.992	10^−7^–10^−2^	8.4 × 10^−8^	[66]
	Si_3_N_4_	APTES-MNP/Co(II)Pc-PAA	-	-	1.3 × 10^−10^–1.3 × 10^−4^	2 × 10^−10^	[67]
Capacitance	ITO	HfO_2_/Co(II)Pc-AP	0.085	0.984	10^−7^–10^−2^	10^−7^	[25]
	*	Biphenylbenzimidazolium	-	0.995	10^−6^–1.8 × 10^−4^	10^−7^	[68]
Optical	*	Paper-based device	-	0.97	4.02 × 10^−8^–1.21 × 10^−7^	3.43 × 10^−8^	[69]
	*	Pyrene-butyrate GUMBOS	-	0.996	2.51 × 10^8^–4.02 × 10^−7^	7.17 × 10^−9^	[70]
	*	oligo (p-phenylenevinylene)	-	0.998	1.67 × 10^−6^–11.1 × 10^−6^	6.54 × 10^−7^	[71]

‘*’: Not applicable, ‘-’: Values not provided.

## Data Availability

The original contributions presented in this study are included in the article/Supplementary Material. Further inquiries can be directed to the corresponding authors.

## Supplementary Materials

The following supporting information can be downloaded at: https://www.mdpi.com/article/10.3390/polym17141937/s1, Table S1. Solubility tests of β-Cyclodextrin derivatives in common solvents ^(a)^. Table S2. Contact angle measurements at 25 °C. Table S3. Equivalent circuit parameters variation as a function of the perchlorate anion concentration (Simulation Errors* in percent are underlined). Table S4. sensitivity and linearity of the (R_ct_/Rc_t0_) and (Q/Q_0_) ration. Table S5. Spiked recoveries and RSDs (%, n = 3) for the determination perchlorate in real water samples by using the developed electrochemical sensor. Figure S1. ^1^H NMR spectra of β-CDBr_7_, β-CDP, in comparison with β-CD (300 MHz, DMSO-d6; * residual DMSO signal; ** residual HOD signal). Figure S2. ^13^C NMR of β-CDP in DMSO_d6. Figure S3. ^31^P NMR of β-CDP in DMSO_d6. Figure S4. FT-IR spectra of β-CD (a), β-CDBr7 (b), and β-CDP (c). Figure S5. TGA thermograms of β-CD, β-CDBr_7_, and β-CDP. Figure S6. DSC thermograms of β-CDP. Figure S7. AFM images of β-CDP on Au substrate. Figure S8. Nyquist diagram for the optimization of the polarization potential of the [Au/β-CDP] structure in PBS (0.01 M) (pH = 7). Figure S9. Cole-Cole impedance spectra of bare and functionalized Au electrode. Figure S10. Screen shot from Zview Software of the simulated (a) impedance and phase spectra and (b) Cole-Cole spectrum using the optimized CEE for 10^−12^ M of perchlorate ions. Figure S11. Evolution of the (R_ct_/R_ct0_) and (Q/Q_0_) ratio determined by simulation as function of cologarithm of ClO_4_^-^ concentration.
