# Supplementary material for: Phosphonium Salt-Functionalized β-Cyclodextrin Film for Ultrasensitive and Selective Electrochemical Impedance Spectroscopy Detection of Perchlorate in Drinking Water
Source: Polymers (Basel). 2025 Jul 15;17(14):1937. doi: 10.3390/polym17141937 (PMC12299713; doi:10.3390/polym17141937)
Supplement: Supplementary file 1 [file polymers-17-01937-s001.zip › polymers-3674472-supplementary.pdf]

Supplementary data

Phosphonium Salt-Functionalized β-Cyclodextrin Film for Ultrasensitive and Selective Electrochemical Impedance Spectroscopy Detection of Perchlorate in Drinking Water

Zeineb Baatout [1], Achref Jebnouni [2*], Nawfel Sakly [1,3],Safa Teka [4*], Nuzaiha Mohamed [5], Sayda Osman [5], Raoudha Soury [4], Mabrouka El Oudi [4], Salman Hamdan Alsaqri [2], Nejmeddine Smida Jaballah [6,1], Mustapha Majdoub[1].

[1] Laboratory of Interfaces and Advanced Materials, University of Monastir, Faculty of Sciences, Monastir, Tunisia

[2] College of Nursing, Medical Surgical Nursing Department, University of Hail, Hai'l KSA

[3] Higher Institute of Applied Sciences and Technology of Mahdia, Rejich, Mahdia, Tunisia

[4] College of Science, Department of Chemistry, University of Hail, Hai'l KSA

[5] College of Public Health, Department of Public Health, University of Hail, Hai'l KSA

[6] Chemistry Department, College of Science and Humanities, Al Quwayiyah, Shaqra University, KSA

* Correspondence: e-mail@e-mail.com; Achrefjebnouni1@gmail.com, safateka@gmail.com

Table S.1. Solubility tests of β-Cyclodextrin derivatives in common solvents [(a)].

	DMF	$CHCl_3$	CH_2Cl_2	Acetone	ethyl Acetate	THF	EtOH	MeOH	Et_2O	Hexane	Water
**β-CD**	++	-	-	-	-	-	-	-	-	-	+
**β-CDBr$_7$**	++	-	-	-	-	-	-	-	-	-	-
**β-CDP**	++	+	±	+	-	++	++	++	-	-	-

[(a)] Solubility was evaluated using 2 mg of product in 1 mL of solvent.

++: Soluble; +: Soluble with heating; ± : Partially soluble ; - Insoluble even with heating.

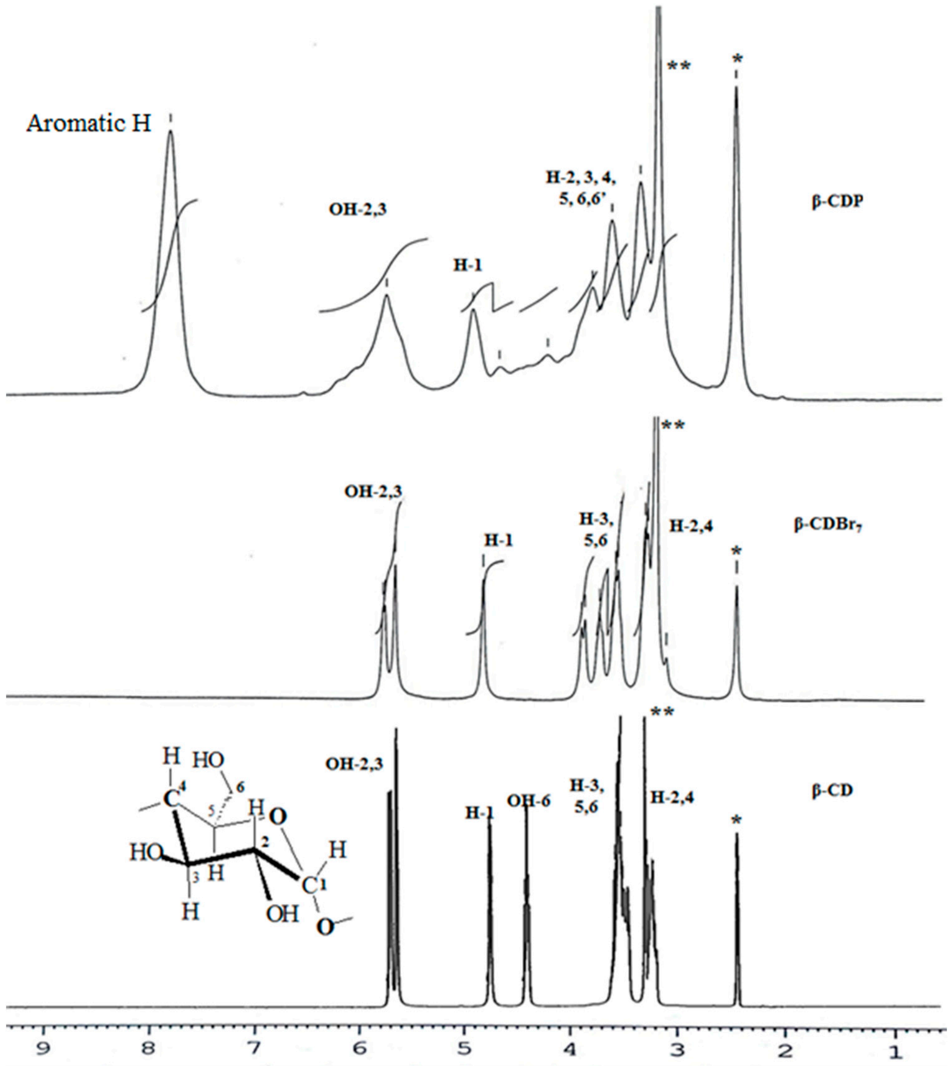

Fig. S.1. ^{1}H NMR spectra of **β-CDBr$_7$**, **β-CDP**, in comparison with **β-CD** (300 MHz, DMSO-d6; * residual DMSO signal; ** residual HOD signal).

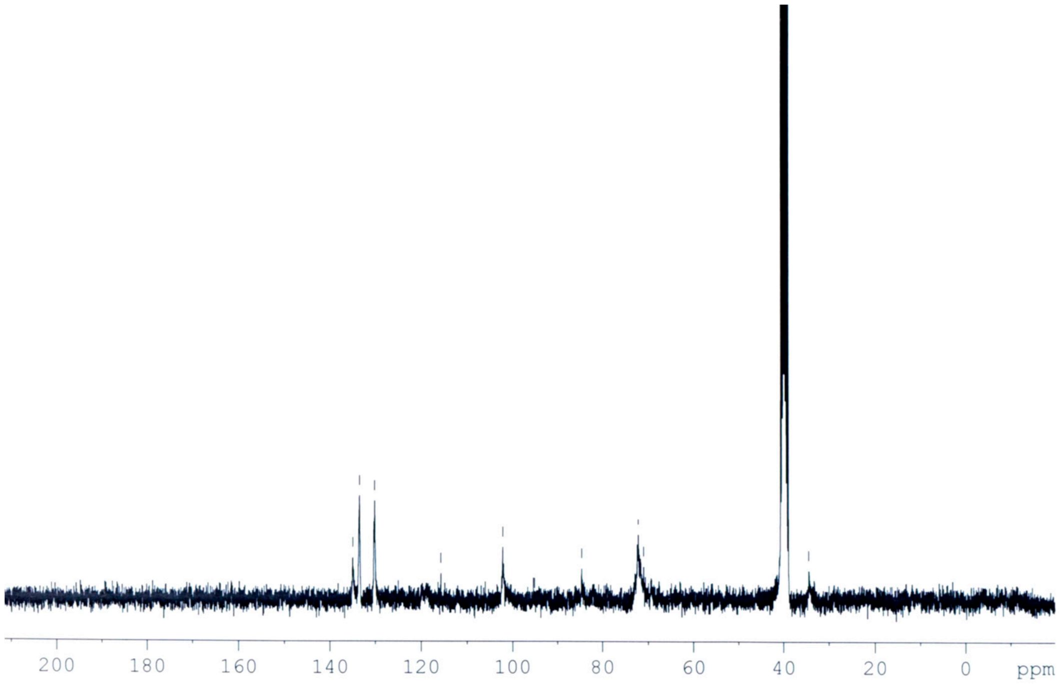

Fig. S.2. ^{13}C NMR of β-CDP in DMSO_d6.

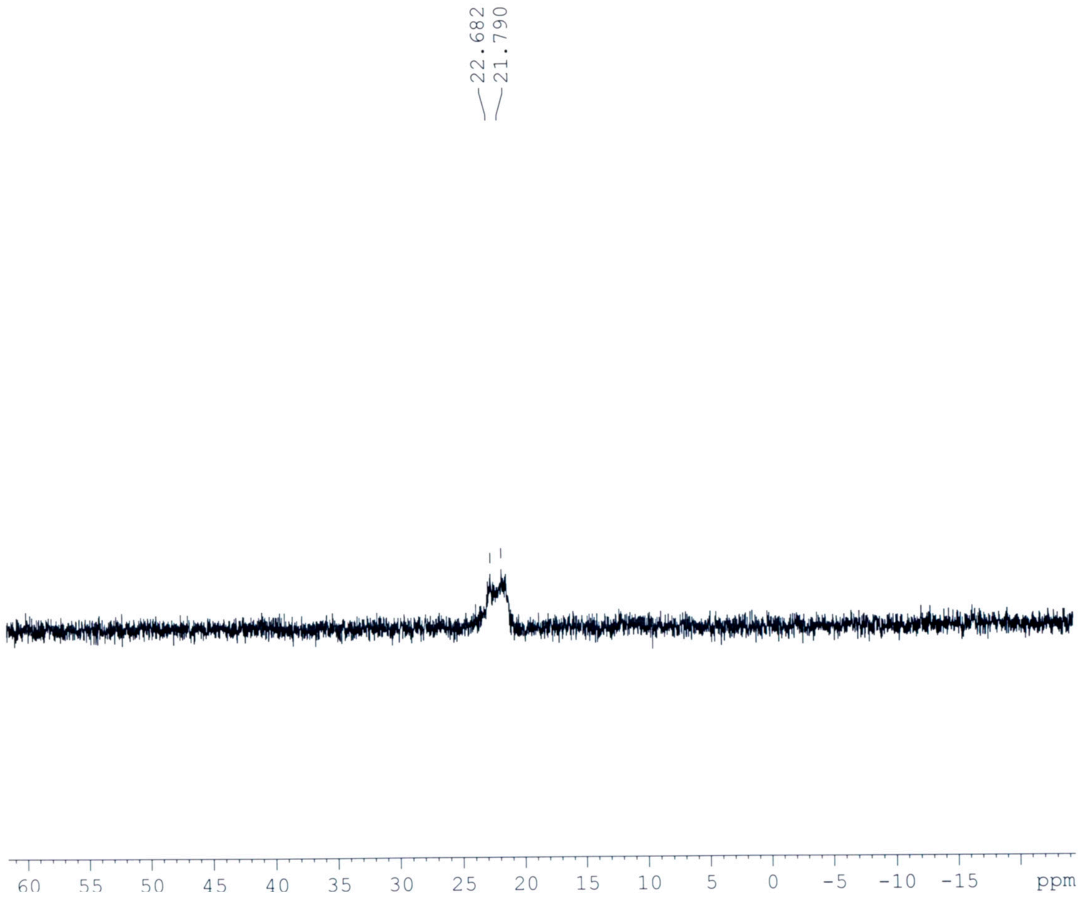

Fig. S.3. ^{31}P NMR of **β-CDP** in DMSO_d6.

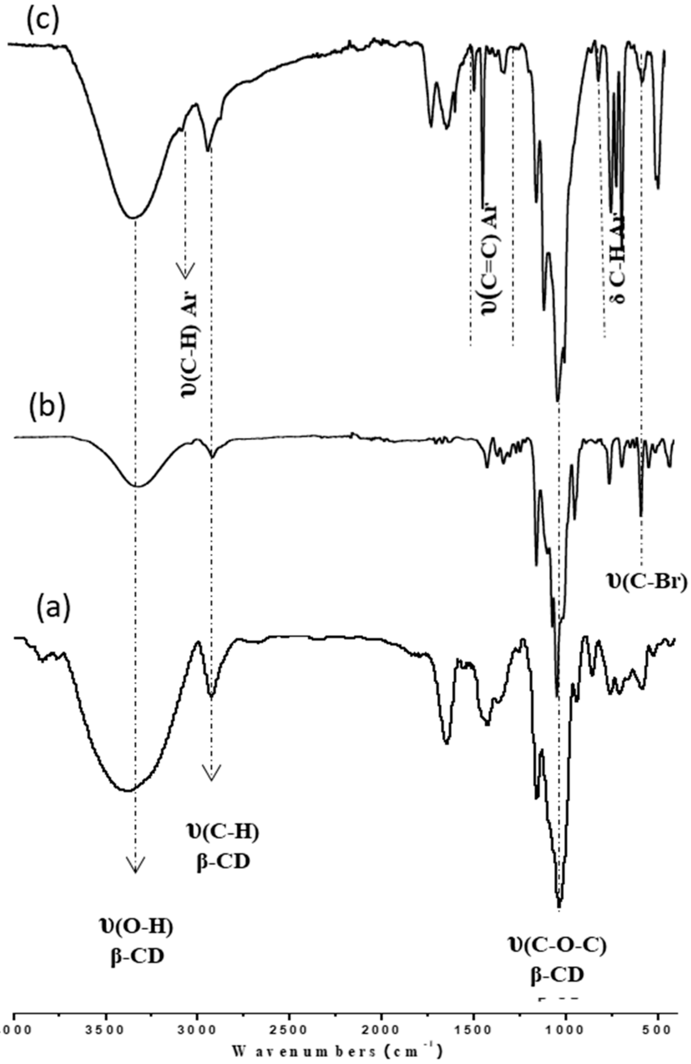

Fig. S.4. FT-IR spectra of **β-CD(a), β-CDBr7 (b),** and **β-CDP(c)**.

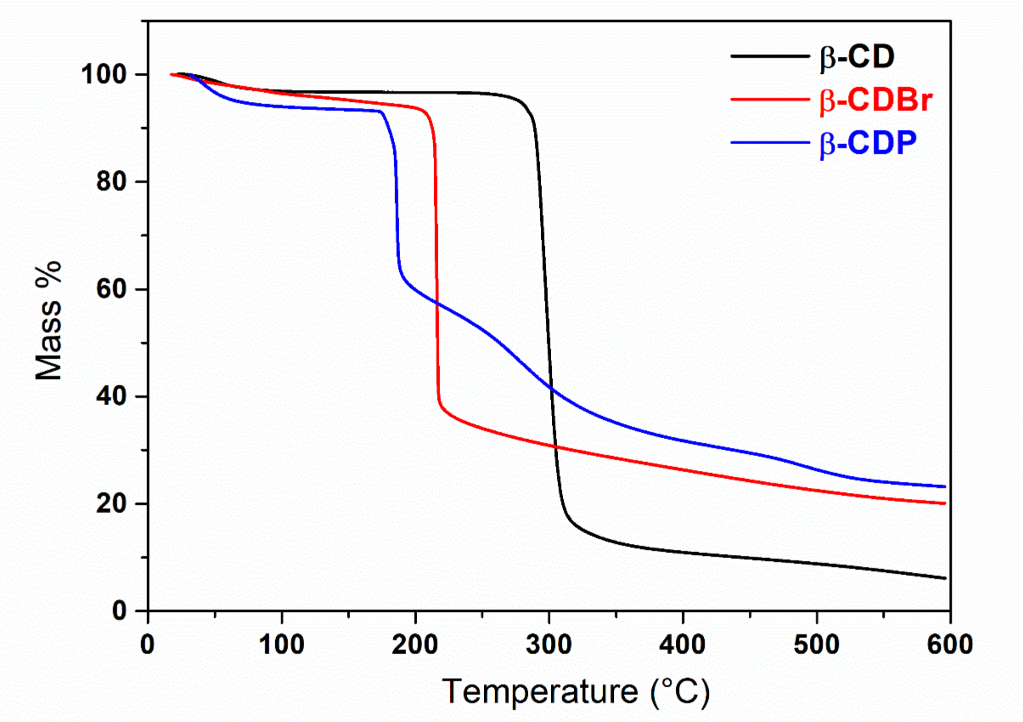

Fig. S.5. TGA thermograms of **β-CD, β-CDBr**$_7$, and **β-CDP**.

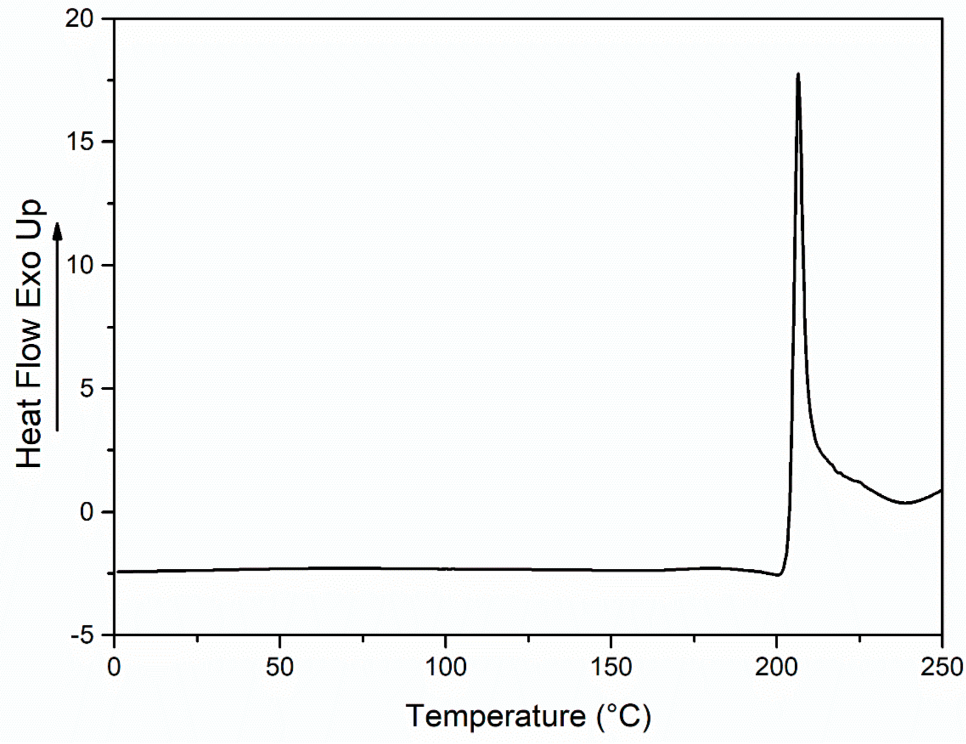

Fig. S.6. DSC thermograms of **β-CDP**.

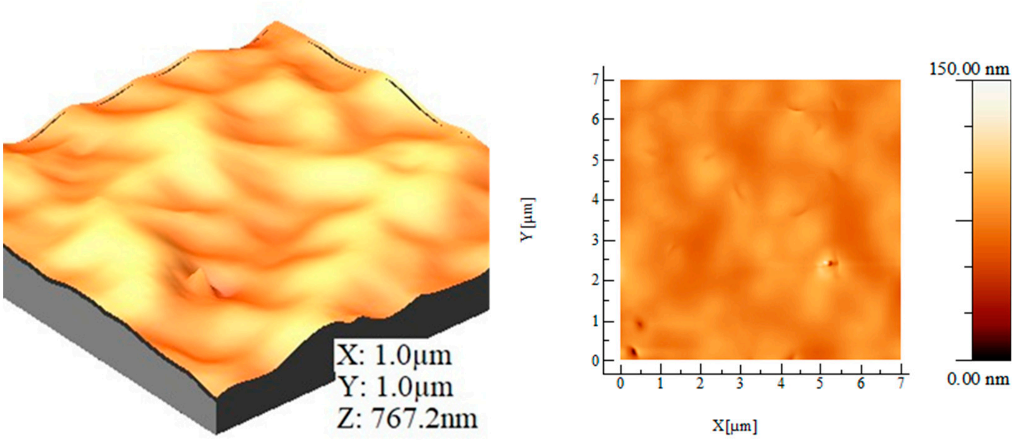

Fig. S.7. AFM images of **β-CDP** on Au substrate.

Table S.2. Contact angle measurements at 25 ° C.

	**Θ° (Water)**	**γ^+ (mJ,m^{-2})**	**γ- (mJ,m^{-2})**	**γ^{AB} (mJ,m^{-2})**
**Gold surface**	70.0	3.8	3.44	7.2
**β-CD** (Liu et al., 2022)	46.4	0.0	35.0	2.0
**β-CDP**	73.0	0.2	8.3	2.6

γ+: acid energy component; γ-: basic energy component;

γ^{AB}: polar energy.

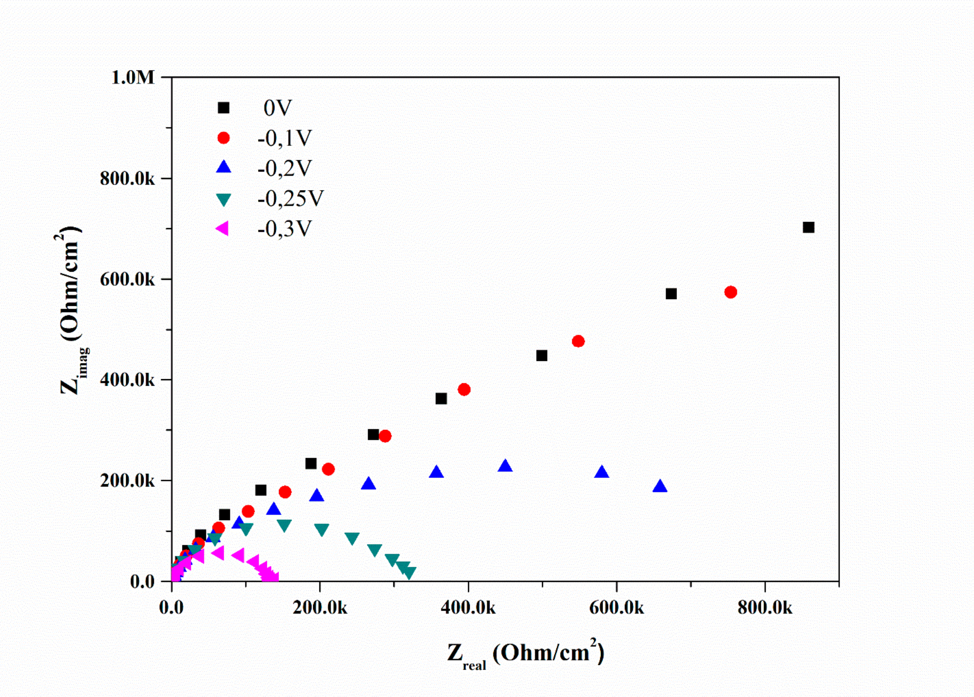

Fig. S.8. Nyquist diagram for the optimization of the polarization potential of the [Au/**β-CDP**] structure in PBS (0.01 M) (pH = 7).

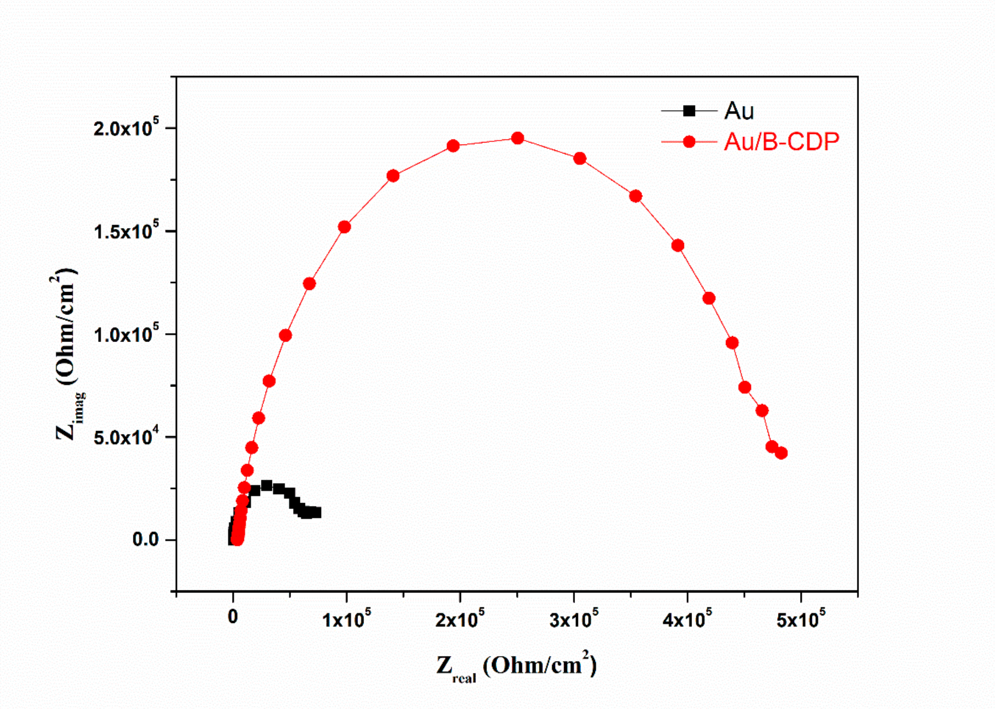

Fig. S.9. Cole-Cole impedance spectra of bare and functionalized Au electrode

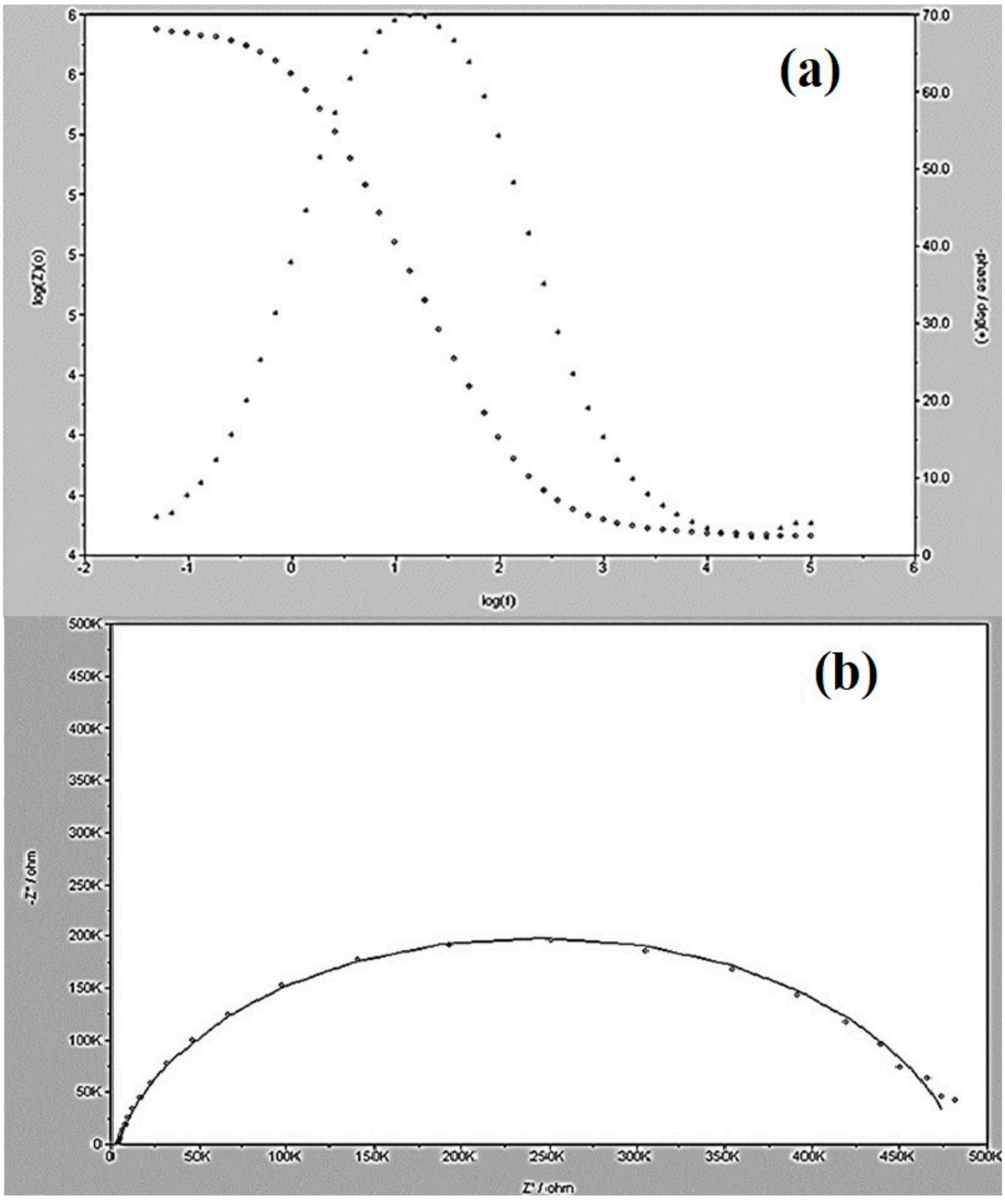

Fig. S.10. Screen shot from Zview Software of the simulated (a) impedance and phase spectra and (b) Cole-Cole spectrum using the optimized CEE for 10^{-12} M of perchlorate ions.

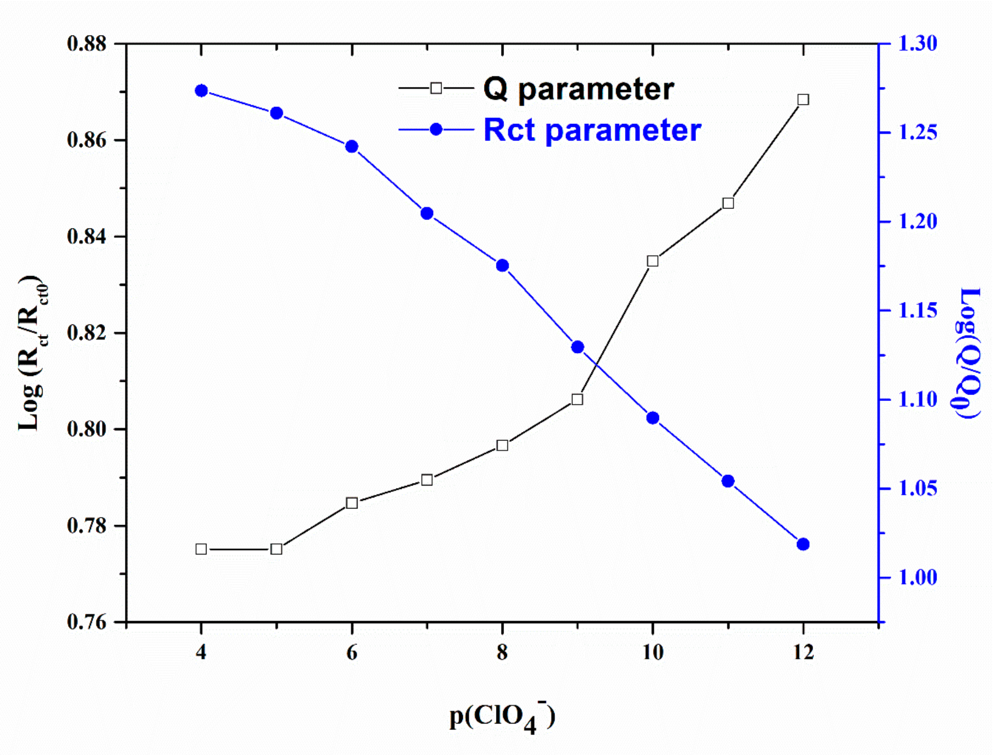

Fig.S.11. Evolution of the (R_{ct}/R_{ct0}) and (Q/Q_0) ratio determined by simulation as function of cologarithm of ClO_4^- concentration

Table S.3. Equivalent circuit parameters variation as a function of the perchlorate anion concentration (Simulation Errors* in percent are underlined)

p[ClO_4^-]	R_s (KΩ)	R_{ct}(KΩ)	CPE (µF)	n	χ2
-	3.920 ± 0.773%	479± 1.157%	0.418± 1.192%	0.880 ± 0.427%	0.03044
12	3.330 ± 0.517%	488± 0.737%	0.363± 1.252%	0.893 ± 0.266%	0.00174
11	3.210 ± 0.502%	505± 0.713%	0.354± 1.200%	0.897 ± 0.253%	0.00163
10	3.150 ± 0.501%	522± 0.714%	0.349± 1.186%	0.899 ± 0.250%	0.00163
9	2.845 ± 0.503%	541± 0.708%	0.337± 1.160%	0.904 ± 0.241%	0.00150
8	2.746± 0.485%	563± 0.683%	0.333± 1.102%	0.905 ± 0.228%	0.00161
7	2.647± 0.489%	577± 0.688%	0.330± 1.098%	0.906 ± 0.226%	0.00151
6	2.393± 0.512%	595± 0.716%	0.328± 1.119%	0.907 ± 0.228%	0.00162
5	2.321± 0.521%	604± 0.726%	0.324± 1.130%	0.909 ± 0.229%	0.00167
4	2.287± 0.528%	610± 0.738%	0.324± 1.140%	0.909 ± 0.231%	0.00172

* **Errors** refer to the differences between the simulated values and the experimental measurements.

Table S.4. sensitivity and linearity of the (R_{ct}/Rc_{t0}) and (Q/Q_0) ration

Ratio	Sensitivity	Linearity (%)
**R_{ct}/R_{ct0}**	0.03365	98.408
**Q/Q_0**	0.01176	91.567

Table S.5. Spiked recoveries and RSDs (%, n=3) for the determination perchlorate in real water samples by using the developed electrochemical sensor.

Sample	Added (nM)	Found (nM)	Recovery* ± RSD(%)
Botteled Drinking Water 01	0.5	0.529	105.8 ± 4.6
	50	48.458	96.9 ± 3.9
	500	492.6	98.5 ± 2.4
Botteled Drinking water 02	0.5	0.521	104.3 ± 3.9
	50	48.76	97.5 ± 3.1
	500	494.2	98.8 ± 2.8
Tape water	0.5	0.549	109.8 ± 4.8
	50	53.553	107.1 ± 3.7
	500	490.5	98.1 ± 2.6
Home tape water	0.5	0.539	107.8 ± 4.2
	50	53.771	107.5 ± 4.1
	500	492.8	98.5 ± 3.2

*Average value from three independent experiments.
